# Comparative In Vitro Assessment of the Antimicrobial Properties of Irreversible Hydrocolloid Impression Material Modified with Two Different Nanoparticles

**DOI:** 10.7759/cureus.114345

**Published:** 2026-08-11

**Authors:** Hima Bindu G, Youshitha B, Vivek Anne, Tejosmita Chowdary Pavuluri, Y Vijaya Lakshmi, Moulinath Chikati Deepak

**Affiliations:** 1 Department of Prosthodontics, CKS Teja Institute of Dental Sciences & Research, Tirupathi, IND; 2 Department of Dentistry, Always Smile Dental Care, North Brunswick Township, USA; 3 Department of Dentistry, Rutgers Biomedical and Health Sciences, Newark, USA; 4 Department of Prosthodontics, KGF College of Dental Sciences &amp; Hospital, Kolar, IND

**Keywords:** alginate, antimicrobial agents, dental impression materials, nanoparticles, streptococcus mutans, titanium dioxide, zirconium dioxide

## Abstract

Background

Dental impressions are frequently contaminated with oral microorganisms during clinical procedures, increasing the risk of cross-contamination between patients, clinicians, and dental laboratory personnel. Incorporating antimicrobial nanoparticles into impression materials may provide an alternative to conventional disinfection methods. This in vitro study evaluated and compared the antimicrobial efficacy of irreversible hydrocolloid impression material modified with titanium dioxide (TiO₂) and zirconium dioxide (ZrO₂) nanoparticles against *Streptococcus mutans*.

Methodology

A total of 120 disc-shaped specimens were prepared and allocated into five groups (n = 20): control, 2% TiO₂, 5% TiO₂, 2% ZrO₂, and 5% ZrO₂. Antimicrobial activity was assessed using the agar diffusion method by measuring the zone of inhibition after 24 hours of incubation. Data were analyzed using descriptive statistics, one-way analysis of variance (ANOVA), and Tukey's post hoc test (p ≤ 0.05).

Results

The control group exhibited no antimicrobial activity. Incorporation of both nanoparticles significantly increased the zone of inhibition (p < 0.001). The highest antimicrobial activity was observed in the 5% TiO₂ group (5.75 ± 0.43 mm), followed by 2% TiO₂ (4.98 ± 0.28 mm), 5% ZrO₂ (3.48 ± 0.28 mm), and 2% ZrO₂ (1.73 ± 0.17 mm). Antibacterial activity increased with nanoparticle concentration, and TiO₂ demonstrated significantly greater efficacy than ZrO₂.

Conclusions

Both TiO₂ and ZrO₂ nanoparticles enhanced the antimicrobial activity of irreversible hydrocolloid impression material, with TiO₂ showing superior efficacy. Nanoparticle incorporation represents a promising strategy for developing self-disinfecting impression materials; however, further investigations are needed before routine clinical application.

## Introduction

Oral health is integral to overall well-being, with teeth playing essential roles in mastication, speech, aesthetics, and maintenance of oral function. Loss of teeth due to dental caries, periodontal disease, trauma, or other conditions can result in partial or complete edentulism, necessitating prosthodontic rehabilitation. Successful fabrication of complete dentures, removable partial dentures, fixed prostheses, and implant-supported restorations depends on obtaining an accurate dental impression. An impression is defined as a negative replica of the oral tissues used for the fabrication of a positive cast [1]. The accuracy of an impression is influenced by factors such as tray selection, material manipulation, moisture control, and removal technique, all of which directly affect the fit and longevity of the final prosthesis [2].

Although digital impression systems have gained popularity because of improved patient comfort and streamlined workflows, conventional impression techniques remain widely used due to their affordability, accessibility, and proven accuracy, particularly for complete-arch impressions [3]. Among conventional impression materials, irreversible hydrocolloid (alginate) is one of the most commonly used because of its ease of manipulation, hydrophilic nature, rapid setting time, and cost-effectiveness [4].

Despite these advantages, alginate impressions are highly susceptible to microbial contamination because of their hydrophilic and porous structure. They can retain saliva, blood, and microorganisms, thereby acting as potential vehicles for cross-infection between patients, clinicians, and dental laboratory personnel [5]. Common oral microorganisms isolated from contaminated impressions include *Streptococcus mutans*, *Staphylococcus aureus*, *Candida *species, *Escherichia coli*, and *Pseudomonas *species [6]. Conventional disinfection methods, such as immersion or spraying with glutaraldehyde, iodophors, or phenolic compounds, effectively reduce microbial contamination but may adversely affect the dimensional stability and surface accuracy of alginate impressions [7].

Nanotechnology has emerged as a promising approach for imparting antimicrobial properties to dental materials. Nanoparticles possess a high surface area-to-volume ratio and exhibit broad-spectrum antimicrobial activity while maintaining favorable biological and mechanical properties [8]. Among them, titanium dioxide (TiO₂) and zirconium dioxide (ZrO₂) nanoparticles have demonstrated excellent biocompatibility, chemical stability, and antibacterial efficacy, making them suitable candidates for incorporation into impression materials [9, 10]. However, limited evidence is available regarding their comparative antimicrobial effectiveness when incorporated into irreversible hydrocolloid impression material. Therefore, the aim of this in vitro study was to evaluate and compare the antimicrobial activity of irreversible hydrocolloid impression material incorporated with 2 wt% and 5 wt% TiO₂ and ZrO₂ nanoparticles against *Streptococcus mutans*, to determine the concentration-dependent antimicrobial effect, and to compare the relative efficacy of the two nanoparticle types. The null hypothesis was that the incorporation of TiO₂ and ZrO₂ nanoparticles at different concentrations would not significantly influence the antimicrobial activity of irreversible hydrocolloid impression material against *Streptococcus mutans* and that no significant difference would exist between the antimicrobial efficacy of the two nanoparticle types.

## Materials and methods

This in vitro experimental study was conducted in the Department of Prosthodontics, CKS Teja Institute of Dental Sciences & Research, Tirupati, India, after obtaining approval from the Institutional Ethics Committee (Approval No. CKS/PROSTHO/23-24/03). Sample size estimation was performed a priori using G*Power software (version 3.1.9.7, Heinrich Heine University, Düsseldorf, Germany) based on data from a previous study [11]. A total of 120 specimens were included in the study.

**Figure 1:**
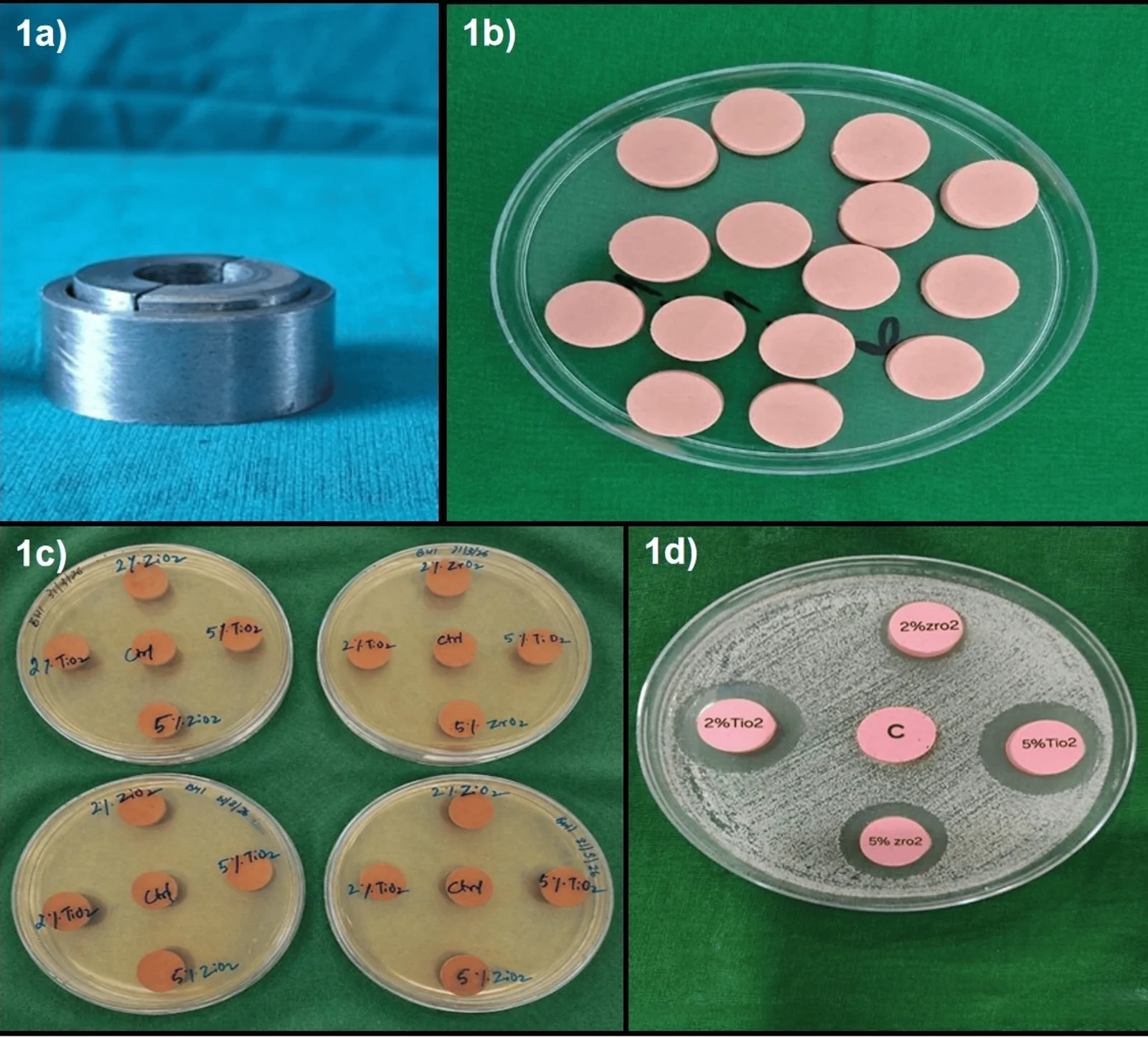
Experimental procedure and representative antimicrobial evaluation (a) Standardized stainless-steel mold used for specimen fabrication. (b) Prepared disc-shaped irreversible hydrocolloid specimens. (c) Agar diffusion assay showing the experimental groups after incubation. (d) Representative inhibition zones produced by the control, 2 wt% titanium dioxide (TiO₂), 5 wt% TiO₂, 2 wt% zirconium dioxide (ZrO₂), and 5 wt% ZrO₂ specimens against *Streptococcus mutans*.

A standardized stainless-steel mold consisting of two cylindrical components measuring 30 mm in internal diameter × 16 mm in height and 15 mm in internal diameter × 19 mm in height was used for specimen fabrication (Figure 1a). The irreversible hydrocolloid impression material (Algitex®, Dental Products of India (DPI), a division of The Bombay Burmah Trading Corporation Ltd., Rudrapur, Uttarakhand, India) was modified by incorporating TiO₂ nanoparticles (NRL, Startup India; CAS No. 13463-67-7; purity 99.5%; particle size 30-50 nm) or ZrO₂ nanoparticles (NRL, Startup India; CAS No. 1314-23-4; purity 99.5%; particle size 30-50 nm) at concentrations of 2 wt% and 5 wt%. The selected concentrations were based on previous studies evaluating nanoparticle incorporation into alginate impression materials [12, 13]. The required quantity of nanoparticle powder was weighed separately and dry-mixed with the alginate powder by tumbling to obtain a homogeneous distribution.

The modified and unmodified powders were mixed with deionized water (CloudGarden Ultra Pure Demineralized Water, Hyderabad, India) according to the manufacturer's instructions. The mixed material was loaded into the larger mold positioned on a glass slab. The smaller mold was then pressed centrally into the material until excess material extruded from the mold. A flat glass plate was placed over the mold, excess material was removed, and the material was allowed to set completely. After setting, the specimens were carefully retrieved. Disc-shaped specimens measuring 15 mm in diameter and 3 mm in thickness were prepared for antimicrobial evaluation (Figure 1b).

Grouping of specimens

A total of 120 specimens were prepared according to the study design and equally distributed into five groups (n = 20) (Figure 1c). They are as follows: Control: conventional irreversible hydrocolloid impression material; 2% ZrO₂: alginate containing 2 wt% ZrO₂ nanoparticles; 5% ZrO₂: alginate containing 5 wt% ZrO₂ nanoparticles; 2% TiO₂: alginate containing 2 wt% TiO₂ nanoparticles; 5% TiO₂: alginate containing 5 wt% TiO₂ nanoparticles

Antimicrobial assessment

*Streptococcus mutans* was selected as the test microorganism because it is a well-established cariogenic bacterium commonly found in dental plaque and saliva and is widely used for evaluating the antimicrobial activity of dental materials. The antimicrobial evaluation was performed in the Department of Microbiology, Saveetha Dental College and Hospitals, Saveetha Institute of Medical and Technical Sciences (SIMATS), Chennai, India. A laboratory-maintained strain of *Streptococcus mutans* was used for antimicrobial assessment by the agar diffusion method. Brain heart infusion (BHI) agar plates were inoculated with lawn cultures of *Streptococcus mutans*, and the prepared specimens were aseptically placed onto the agar surface. The plates were incubated at 37°C for 24 hours. Following incubation, the antimicrobial activity of each specimen was assessed by measuring the diameter of the zone of inhibition in millimeters (Figure 1d).

Statistical analysis

Data analysis was performed with IBM SPSS Statistics, Version 27.0 (IBM Corp., Armonk, NY, USA). Descriptive statistics were generated, and group comparisons were made using one-way analysis of variance (ANOVA) with Tukey’s post hoc test. Significance was set at p < 0.05.

## Results

The descriptive statistics are presented in Table 1. The control group exhibited no antimicrobial activity (0.00 ± 0.00 mm). Incorporation of both TiO₂ and ZrO₂ nanoparticles significantly increased the zone of inhibition compared with the control. Among all experimental groups, the highest antimicrobial activity was observed in the 5% TiO₂ group (5.75 ± 0.43 mm), followed by the 2% TiO₂ group (4.98 ± 0.28 mm). The ZrO₂ groups also demonstrated concentration-dependent antimicrobial activity, with mean inhibition zones of 1.73 ± 0.17 mm and 3.48 ± 0.28 mm for the 2% and 5% concentrations, respectively.

**Table 1:** Descriptive statistics of antimicrobial activity of all study groups; Total specimens = 120 (20 specimens in each group) TiO₂: titanium dioxide nanoparticles; ZrO₂: zirconium dioxide nanoparticles

Group	n	Mean ± SD (mm)	Minimum	Maximum	Median
Control	40	0.00 ± 0.00	0.00	0.00	0.00
2% TiO₂	20	4.98 ± 0.28	4.50	5.50	4.95
5% TiO₂	20	5.75 ± 0.43	5.00	6.50	5.75
2% ZrO₂	20	1.73 ± 0.17	1.50	2.00	1.70
5% ZrO₂	20	3.48 ± 0.28	3.00	4.00	3.45

One-way ANOVA revealed statistically significant differences among the TiO₂ groups (F = 2225.29, p < 0.001) and the ZrO₂ groups (F = 1674.75, p < 0.001), indicating that nanoparticle incorporation significantly influenced the antimicrobial activity of the impression material (Table 2).

**Table 2:** One-way ANOVA comparing antimicrobial activity among TiO₂ and ZrO₂ groups TiO₂: titanium dioxide nanoparticles; ZrO₂: zirconium dioxide nanoparticles; *Statistically significant (p < 0.001).

Nanoparticle	Sum of Squares (Between)	Sum of Squares (Within)	df	F-value	p-value
TiO₂	330.53	4.17	2.57	2225.29	<0.001*
ZrO₂	124.88	2.13	2.57	1674.75	<0.001*

Tukey's post hoc analysis demonstrated statistically significant differences between all pairwise comparisons (p < 0.001), indicating that increasing the concentration of both TiO₂ and ZrO₂ nanoparticles significantly enhanced antimicrobial activity (Table 3).

**Table 3:** Tukey's post hoc comparison of antimicrobial activity among study groups TiO₂: titanium dioxide nanoparticles; ZrO₂: zirconium dioxide nanoparticles; *Statistically significant (p < 0.001).

Comparison	Mean Difference (mm)	95% Confidence Interval	p-value
Control vs 2% TiO₂	4.98	4.84–5.12	<0.001*
Control vs 5% TiO₂	5.75	5.57–5.93	<0.001*
2% TiO₂ vs 5% TiO₂	0.77	0.59–0.95	<0.001*
Control vs 2% ZrO₂	1.73	1.63–1.83	<0.001*
Control vs 5% ZrO₂	3.48	3.32–3.64	<0.001*
2% ZrO₂ vs 5% ZrO₂	1.75	1.61–1.89	<0.001*

In a direct comparison between the two nanoparticles at both concentrations, TiO₂ nanoparticles demonstrated greater antimicrobial activity than ZrO₂ nanoparticles. The difference was 3.25 mm at the 2% concentration and 2.27 mm at the 5% concentration. Overall, the 5% TiO₂ group exhibited the highest antibacterial activity among all experimental groups (Table 4).

**Table 4:** Comparative antimicrobial activity of TiO₂ and ZrO₂ nanoparticles TiO₂: titanium dioxide nanoparticles; ZrO₂: zirconium dioxide nanoparticles

Concentration	TiO₂ (Mean ± SD, mm)	ZrO₂ (Mean ± SD, mm)	Mean Difference (mm)
2%	4.98 ± 0.28	1.73 ± 0.17	3.25
5%	5.75 ± 0.43	3.48 ± 0.28	2.27

## Discussion

Dental impressions can act as a source of microbial transmission from the oral cavity to clinicians, dental auxiliaries, and laboratory personnel. Consequently, considerable research has focused on developing impression materials with inherent antimicrobial properties that minimize cross-contamination without requiring additional disinfection procedures. In this context, the present investigation evaluated the antimicrobial efficacy of irreversible hydrocolloid impression material incorporated with TiO₂ and ZrO₂ nanoparticles against *Streptococcus mutans*.

The results demonstrated that incorporation of both TiO₂ and ZrO₂ nanoparticles significantly enhanced the antimicrobial activity of alginate compared with the unmodified control group. The absence of a zone of inhibition in the control specimens confirms that conventional alginate possesses no intrinsic antibacterial activity, which is consistent with its hydrophilic and porous nature that readily retains microorganisms during impression making [5, 14]. Incorporation of nanoparticles therefore represents a promising strategy for reducing microbial contamination during impression handling and laboratory procedures. The improved antibacterial activity can be attributed to the unique physicochemical characteristics of metal oxide nanoparticles. Their high surface area-to-volume ratio facilitates close interaction with bacterial cell membranes, resulting in membrane disruption and interference with essential cellular functions [9]. Unlike conventional disinfectants that act only after impression removal, nanoparticles incorporated within the impression material may provide continuous antimicrobial activity throughout the material.

Among the tested nanoparticles, TiO₂ demonstrated greater antibacterial efficacy than ZrO₂ at both concentrations. This superior performance is primarily related to its photocatalytic activity, which generates reactive oxygen species (ROS), including hydroxyl radicals and superoxide ions. By generating oxidative stress, these reactive species disrupt bacterial membranes, alter proteins, and degrade nucleic acids, which culminates in the destruction of the bacterial cell [15]. These findings agree with those of Omer et al., who reported a significant concentration-dependent increase in the antibacterial activity of alginate incorporated with TiO₂ nanoparticles against *Streptococcus mutans* [12]. Similarly, Hamouda attributed the antibacterial efficacy of TiO₂ nanoparticles to ROS generation [16], while Lubojanski et al. emphasized that their high surface reactivity and extensive surface area make TiO₂ one of the most promising antimicrobial nanomaterials for dental applications [17].

Beyond their antimicrobial activity, the incorporation of TiO₂ and ZrO₂ nanoparticles may also influence the physicochemical properties of alginate impression materials. Previous studies have reported that nanoparticle incorporation can improve certain mechanical characteristics while maintaining acceptable dimensional stability at appropriate concentrations. However, not all studies have reported uniformly favorable outcomes. Higher nanoparticle concentrations have been associated with alterations in surface detail reproduction, setting characteristics, viscosity, and other mechanical properties, which may be influenced by nanoparticle concentration, particle size, dispersion within the alginate matrix, and experimental methodology [12,18]. Therefore, careful optimization of nanoparticle concentration is essential to achieve enhanced antimicrobial efficacy without compromising the clinical performance of the impression material. Since the present investigation evaluated only antimicrobial activity, further studies are required to assess the influence of nanoparticle incorporation on the physical, mechanical, and dimensional properties of alginate impression materials.

ZrO₂ nanoparticles also exhibited significant antibacterial activity compared with the control group, although their efficacy was lower than that of TiO₂. Their antimicrobial effect has been attributed to disruption of bacterial cell membranes and interference with intracellular metabolic processes rather than ROS generation [19]. Previous studies have similarly reported that zirconia nanoparticles reduce bacterial adhesion and biofilm formation while maintaining excellent biocompatibility and mechanical stability [19,20]. The comparatively lower antibacterial activity observed in the present study is therefore consistent with the known biological behavior of zirconia.

Another important finding was the concentration-dependent increase in antimicrobial activity observed for both nanoparticles. The 5% concentration produced significantly larger inhibition zones than the corresponding 2% concentration, indicating that increasing nanoparticle concentration enhances bacterial inhibition. Similar concentration-dependent responses have been reported by Omer et al. [12] and other studies evaluating nanoparticle-modified dental materials [8, 21]. Increasing nanoparticle concentration provides more active antimicrobial sites and greater interaction with bacterial cells, thereby improving antibacterial efficacy.

Nevertheless, increasing nanoparticle concentration beyond an optimal level may promote particle agglomeration, resulting in non-uniform distribution within the material and potentially affecting both antimicrobial performance and physical properties. Therefore, optimization of nanoparticle concentration remains essential to achieve an appropriate balance between antimicrobial efficacy and the clinical performance of impression materials.

The comparative analysis demonstrated that TiO₂ nanoparticles exhibited significantly greater antimicrobial activity than ZrO₂ nanoparticles at both tested concentrations. This difference is likely related to their distinct mechanisms of antibacterial action. While TiO₂ exerts its effect predominantly through photocatalytic generation of reactive oxygen species, ZrO₂ primarily inhibits bacterial growth by disrupting cell membrane integrity and interfering with intracellular metabolic processes [15, 19]. The stronger oxidative activity of TiO₂ may therefore account for the larger zones of inhibition observed in the present investigation.

From a clinical perspective, incorporation of antimicrobial nanoparticles into irreversible hydrocolloid impression material offers a practical approach for reducing microbial contamination at its source. Conventional disinfection methods rely on spraying or immersion after impression removal, procedures that are technique-sensitive and may influence dimensional stability or surface detail reproduction when not performed appropriately [22]. By imparting intrinsic antimicrobial properties to the impression material, nanoparticle incorporation has the potential to supplement routine infection control measures and reduce the microbial burden during impression handling, transportation, and laboratory processing. However, nanoparticle-modified impression materials should currently be considered an adjunct rather than a replacement for established infection control protocols until their long-term clinical performance has been validated.

A major strength of this investigation is the direct comparison of two clinically relevant metal oxide nanoparticles incorporated into the same irreversible hydrocolloid impression material under standardized experimental conditions. The inclusion of two concentrations for each nanoparticle enabled evaluation of concentration-dependent antibacterial activity, providing useful information regarding their relative antimicrobial performance. Furthermore, the use of *Streptococcus mutans*, one of the principal cariogenic microorganisms in the oral cavity, enhances the clinical relevance of the findings.

Despite these encouraging findings, certain limitations should be acknowledged. The present investigation was conducted under in vitro conditions using only a single bacterial species (*Streptococcus mutans*), which cannot fully replicate the complex polymicrobial environment of the oral cavity. Furthermore, the antimicrobial efficacy was assessed exclusively using the agar diffusion method, which primarily evaluates the diffusion of antimicrobial agents through the culture medium and may not accurately reflect the antibacterial performance of nanoparticle-modified impression materials under clinical conditions. Another limitation was the absence of a conventional disinfectant-treated control, which would have provided a clinically relevant benchmark for comparison. In addition, only the antimicrobial activity of the modified impression material was evaluated, whereas clinically important properties such as dimensional stability, surface detail reproduction, tear strength, setting characteristics, compatibility with gypsum products, and the effect of nanoparticle incorporation on handling characteristics were not investigated. The possibility of nanoparticle agglomeration and its influence on the uniform distribution of nanoparticles within the alginate matrix was also not assessed. Future studies should evaluate these physical and mechanical properties alongside antimicrobial efficacy using complementary methods such as direct contact and colony-forming unit (CFU) assays, multispecies oral biofilm models, and clinical investigations. Additionally, the inclusion of conventional disinfectant-treated controls, long-term biocompatibility assessment, and evaluation of different nanoparticle concentrations or combinations would provide a more comprehensive understanding of the clinical applicability of nanoparticle-modified impression materials.

## Conclusions

The incorporation of TiO₂ and ZrO₂ nanoparticles significantly enhanced the antimicrobial activity of irreversible hydrocolloid impression material against Streptococcus mutans, with TiO₂ demonstrating greater efficacy than ZrO₂ at both evaluated concentrations. The concentration-dependent antimicrobial effect observed under in vitro conditions suggests that nanoparticle-modified alginate has the potential to serve as a self-disinfecting impression material for improving infection control during impression procedures. However, further investigations are required to evaluate its physical and mechanical properties, biocompatibility, and long-term clinical performance before routine clinical application can be recommended.

## References

[1] (2017). The glossary of prosthodontic terms: ninth edition. J Prosthet Dent.

[2] Hussain MW, Chaturvedi S, Naqash TA, Ahmed AR, Das G, Rana MH, Abdelmonem AM (2020). Influence of time, temperature and humidity on the accuracy of alginate impressions. J Ayub Med Coll Abbottabad.

[3] Ahmed S, Hawsah A, Rustom R (2024). Digital impressions versus conventional impressions in prosthodontics: a systematic review. Cureus.

[4] Hamalian TA, Nasr E, Chidiac JJ (2011). Impression materials in fixed prosthodontics: influence of choice on clinical procedure. J Prosthodont.

[5] Ginjupalli K, Alla RK, Tellapragada C, Gupta L, Upadhya Perampalli N (2016). Antimicrobial activity and properties of irreversible hydrocolloid impression materials incorporated with silver nanoparticles. J Prosthet Dent.

[6] Lemos JA, Palmer SR, Zeng L (2019). The biology of Streptococcus mutans. Microbiol Spectr.

[7] Sivamani Chidambaram R, Rajmohan S, Olive Prasad P, Kalyani D, Mallikarjuna R, Ganiga Channaiah S (2024). Evaluation of the effectiveness of disinfectants on impression materials. Cureus.

[8] Priyadarsini S, Mukherjee S, Mishra M (2018). Nanoparticles used in dentistry: a review. J Oral Biol Craniofac Res.

[9] Gronwald B, Kozłowska L, Kijak K (2023). Nanoparticles in dentistry-current literature review. Coatings.

[10] Serov DA, Gritsaeva AV, Yanbaev FM, Simakin AV, Gudkov SV (2024). Review of antimicrobial properties of titanium dioxide nanoparticles. Int J Mol Sci.

[11] Hamedi Rad F, Ghaffari T, Safavi SH (2010). In vitro evaluation of dimensional stability of alginate impressions after disinfection by spray and immersion methods. J Dent Res Dent Clin Dent Prospects.

[12] Omer RA, Abdel-Rahman HK, Saleh MM, Al-Hawezi SS, Ikram F (2023). Effect of adding titanium dioxide nanoparticles on anti-microbial activity and surface detail reproduction of dental alginate. J Bagh Coll Dent.

[13] Al-Nema LM, Al-Ali AA (2023). Compressive strength of alginate impression material incorporated with silver and zirconium oxide nanoparticles. Al-Rafidain Dent J.

[14] Hardan L, Bourgi R, Cuevas-Suárez CE (2022). Disinfection procedures and their effect on the microorganism colonization of dental impression materials: a systematic review and meta-analysis of in vitro studies. Bioengineering (Basel).

[15] Ramburrun P, Pringle NA, Dube A, Adam RZ, D'Souza S, Aucamp M (2021). Recent advances in the development of antimicrobial and antifouling biocompatible materials for dental applications. Materials (Basel).

[16] Mohamed Hamouda I (2012). Current perspectives of nanoparticles in medical and dental biomaterials. J Biomed Res.

[17] Lubojanski A, Dobrzynski M, Nowak N (2021). Application of selected nanomaterials and ozone in modern clinical dentistry. Nanomaterials (Basel).

[18] Abdel-Rahman HK, Omer RA, Saleh MM, Al-Hawezi SS, Ikram FS (2022). Effect of adding titanium dioxide nanoparticles on some mechanical properties of dental alginate. Sulaimani Dent J.

[19] Bannunah AM (2023). Biomedical applications of zirconia-based nanomaterials: challenges and future perspectives. Molecules.

[20] Khan M, Shaik MR, Khan ST (2020). Enhanced antimicrobial activity of biofunctionalized zirconia nanoparticles. ACS Omega.

[21] Moraes G, Zambom C, Siqueira WL (2021). Nanoparticles in dentistry: a comprehensive review. Pharmaceuticals (Basel).

[22] Allen EP, Bayne SC, Brodine AH, Cronin RJ Jr, Donovan TE, Kois JC, Summitt JB (2001). Annual review of selected dental literature: report of the committee on scientific investigation of the American Academy of Restorative Dentistry. J Prosthet Dent.

